# Supplementary material for: Identification of photoperiod-induced specific miRNAs in the adrenal glands of Sunite sheep (Ovis aries)
Source: Front Vet Sci. 2022 Jul 22;9:888207. doi: 10.3389/fvets.2022.888207 (PMC9354845; doi:10.3389/fvets.2022.888207)
Supplement: Supplementary file 1 [file Data_Sheet_1.ZIP › Supplementary file/miRDeep2 file.zip!/miRDeep2 file/Novel_Structure/SP42A2/Novel_434.pdf]

Provisional ID	:	NC_019458.2_1553
Score total	:	56.6
Score for star read(s)	:	3.9
Score for read counts	:	49.1
Score for mfe	:	2
Score for randfold	:	1.6
Score for cons. seed	:	
Total read count	:	108
Mature read count	:	83
Loop read count	:	0
Star read count	:	25

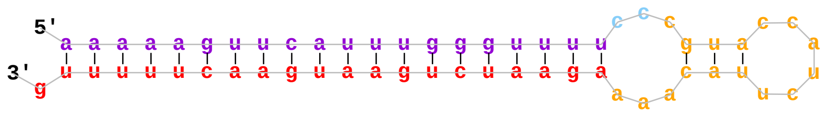

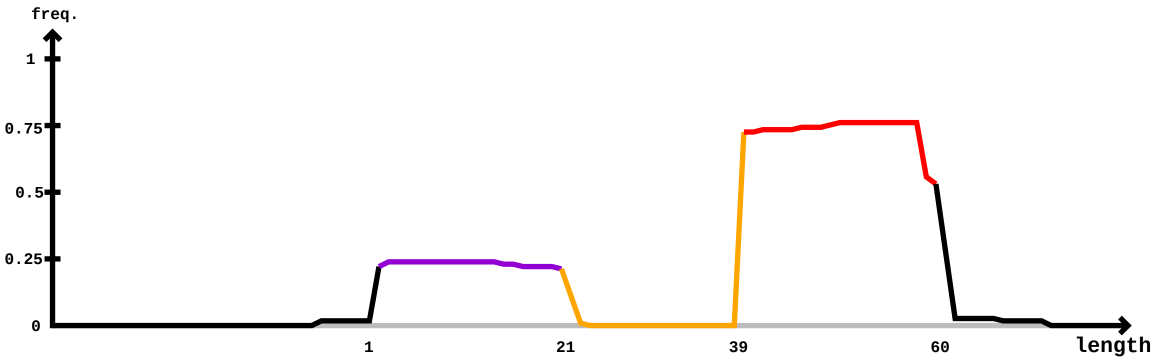

Star **Mature**

```
5'- caaaaugaaugaacuaugauauugcguuggccaaaaaguucauuuggguuuucccguaccaucuuacaaaagaaucugaaugaacuuuuuggccaacccaauacauucau  -3'  obs
    caaaaugaaugaacuaugauauugcguuggccaaaaaguucauuuggguuuucccguaccaucuuacaaaagaaucugaaugaacuuuuuggccaacccaauacauucau       exp
    ....((((((........(((((.((((((((((((((((((((((((((((...(((......)))...)))))))))))))))))))))))))))).)))))))))))   reads   mm   sample
    ..........................uuggccaaaaagCucauuu.................................................................   1       1    seq
    ..........................uuggccGaaaaguucauuugg...............................................................   1       1    seq
    ................................aaaaaguucGuuuggguuuu..........................................................   23      1    seq
    .................................Uaaaguucauuuggguuu...........................................................   1       1    seq
    .................................aaaaguucauuuggguuuuUc........................................................   1       1    seq
    ......................................................................aAaaucugaaugaacuuuu.....................   23      1    seq
    ......................................................................agaaucugaaugaacuuuuu....................   2       0    seq
    ......................................................................aAaaucugaaugaacuuuuug...................   57      1    seq
    ........................................................................aaucugaaugaacuuuuu....................   1       0    seq
    ............................................................................uAaaugaacuuuuuggccaac.............   1       1    seq
    ...............................................................................augaacuuuuuggccaacccaGu........   1       1    seq
    ................................................................................ugaacuuuuuggccaacccaaC........   1       1    seq
```
